# Ifenprodil and Flavopiridol Identified by Genomewide RNA Interference Screening as Effective Drugs To Ameliorate Murine Acute Lung Injury after Influenza A H5N1 Virus Infection

**DOI:** 10.1128/mSystems.00431-19

**Published:** 2019-12-10

**Authors:** Cong Zhang, Yuqing Zhang, Yuhao Qin, Qingchao Zhang, Qiang Liu, Daozhen Shang, Huijun Lu, Xiao Li, Congzhao Zhou, Fengming Huang, Ningyi Jin, Chengyu Jiang

**Affiliations:** a Hefei National Laboratory for Physical Sciences at the Microscale and School of Life Sciences, University of Science and Technology of China, Hefei, Anhui, China; b State Key Laboratory of Biotherapy/Collaborative Innovation Center for Biotherapy, West China Hospital, Sichuan University, Chengdu, China; c State Key Laboratory of Medical Molecular Biology, Institute of Basic Medical Sciences Chinese Academy of Medical Sciences, Department of Biochemistry, School of Basic Medicine Peking Union Medical College, Beijing, China; d Genetic Engineering Laboratory, Institute of Military Veterinary Medicine, Academy of Military Medical Sciences, Changchun, China; Princeton University

**Keywords:** H5N1, lung injury, ifenprodil, flavopiridol, drug repurposing, genomewide RNAi, H5N1

## Abstract

Due to the limitations of effective treatments, avian influenza A H5N1 virus is the most lethal influenza virus strain that causes severe acute lung injury (ALI). To develop effective drugs ameliorating H5N1-induced ALI, we explore an RNA interference (RNAi) screening method to monitor changes in cell death induced by H5N1 infection. We performed RNAi screening on 19,424 genes in A549 lung epithelial cells and examined cell death induced by H5N1 infection. These screens identified 1,137 host genes for which knockdown altered cell viability by over 20%. DrugBank searches of these 1,137 host genes identified 146 validated druggable target genes with 372 drug candidates. We obtained 104 commercially available drugs with 65 validated target genes and examined their improvement of cell viability following H5N1 infection. We identified 28 drugs that could significantly recover cell viability following H5N1 infection and tested 10 in an H5N1-induced-ALI mouse model. The neurological drug ifenprodil and the anticancer drug flavopiridol markedly decreased leukocyte infiltration and lung injury scores in infected mouse lungs, significantly ameliorated edema in infected mouse lung tissues, and significantly improved the survival of H5N1-infected mice. Ifenprodil is an antagonist of the N-methyl-d-aspartate (NMDA) receptor, which is linked to inflammation and lung injury. Flavopiridol is an inhibitor of cyclin-dependent kinase 4 (CDK4), which is linked to leukocyte migration and lung injury. These results suggest that ifenprodil and flavopiridol represent novel remedies against potential H5N1 epidemics in addition to their proven indications. Furthermore, our strategy for identifying repurposable drugs could be a general approach for other diseases.

**IMPORTANCE** Drug repurposing is a quick and economical strategy for developing new therapies with approved drugs. H5N1 is a highly pathogenic avian influenza virus subtype that can cause severe acute lung injury (ALI) and a high mortality rate due to limited treatments. The use of RNA interference (RNAi) is a reliable approach to identify essential genes in diseases. In most genomewide RNAi screenings, virus replication is the readout of interference. Since H5N1 virus infection could induce significant cell death and the percentage of cell death is associated with virus lethality, we designed a genomewide RNAi screening method to identify repurposable drugs against H5N1 virus with cell death as the readout. We discovered that the neurological drug ifenprodil and the anticancer drug flavopiridol could effectively ameliorate murine ALI after influenza A H5N1 virus infection, suggesting that they might be novel remedies for H5N1 virus-induced ALI in addition to the traditional indications.

## Abstract (precis)

Drug repurposing is a quick and economical strategy for developing new therapies with approved drugs. H5N1 is a highly pathogenic avian influenza virus subtype that can cause severe acute lung injury (ALI) and a high mortality rate due to limited treatments. The use of RNA interference (RNAi) is a reliable approach to identify essential genes in diseases. In most genomewide RNAi screenings, virus replication is the readout of interference. Since H5N1 virus infection could induce significant cell death and the percentage of cell death is associated with virus lethality, we designed a genomewide RNAi screening method to identify repurposable drugs against H5N1 virus with cell death as the readout. We discovered that the neurological drug ifenprodil and the anticancer drug flavopiridol could effectively ameliorate murine ALI after influenza A H5N1 virus infection, suggesting that they might be novel remedies for H5N1 virus-induced ALI in addition to the traditional indications.

## INTRODUCTION

Influenza A virus is the most common type among the viruses that cause influenza epidemics, and influenza A viruses can be divided into various subtypes based on the hemagglutinin and neuraminidase proteins on the viral surface (1). H5N1 is a highly pathogenic avian influenza virus subtype that can cause severe acute lung injury (ALI) in human subjects (2). Infection with H5N1 virus is associated with a 52.79% mortality rate (https://www.who.int/influenza/human_animal_interface/2019_02_12_tableH5N1.pdf?ua=1). However, effective treatments are limited. The use of corticoids is controversial, and extracorporeal membrane oxygenation (ECMO), artificial liver support system (ALSS), and mechanical ventilation are often expensive (3). Thus, there is an urgent and continuous need for novel treatments.

Drug repurposing is a quick and economical strategy to identify new therapeutic uses of approved drugs outside their original scope and is estimated to be the source of approximately 30% of newly approved drugs every year (4). An example of successful drug repurposing is the use of the chemotherapeutic agent azidothymidine as a therapy for HIV in the 1980s (4). Thus, the exploration of an efficient method for drug repurposing is necessary.

RNA interference (RNAi) represents a reliable approach to identify genes important in diseases such as acute myeloid leukemia, epithelial ovarian cancer, and schistosomiasis (5–7). Genomewide RNAi screening has been used to identify host genes involved in the replication of the moderately pathogenic influenza H1N1 virus (8–15). Data mining using public databases such as the DrugBank database and the Therapeutic Target Database (TTD) has been increasingly used to identify candidate drugs that could potentially interact with known validated target genes (16, 17). We combined RNAi screening and DrugBank and TTD target gene searches to develop an effective method for identifying repurposable drugs.

In the current study, we used genomewide RNAi screening to identify host genes that influence host cell viability following H5N1 virus infection. The results showed <10% overlap in the candidate host gene targets between H5N1 and the less lethal H1N1 strain. Next, we screened 1,137 critical host genes in RNAi screens using the DrugBank database and identified 372 approved or clinically investigated drugs (with 146 target genes). Validation experiments performed with 104 commercially available drugs (and 65 target genes) identified 28 candidate drugs that significantly increased the viability of H5N1-infected A549 cells. The two most effective drugs among these candidates in a mouse model of H5N1-induced ALI were ifenprodil, an agent with neurological indications, and flavopiridol, an antineoplastic agent.

## RESULTS

### Genomewide RNAi screening of target host genes.

**FIG 1:**
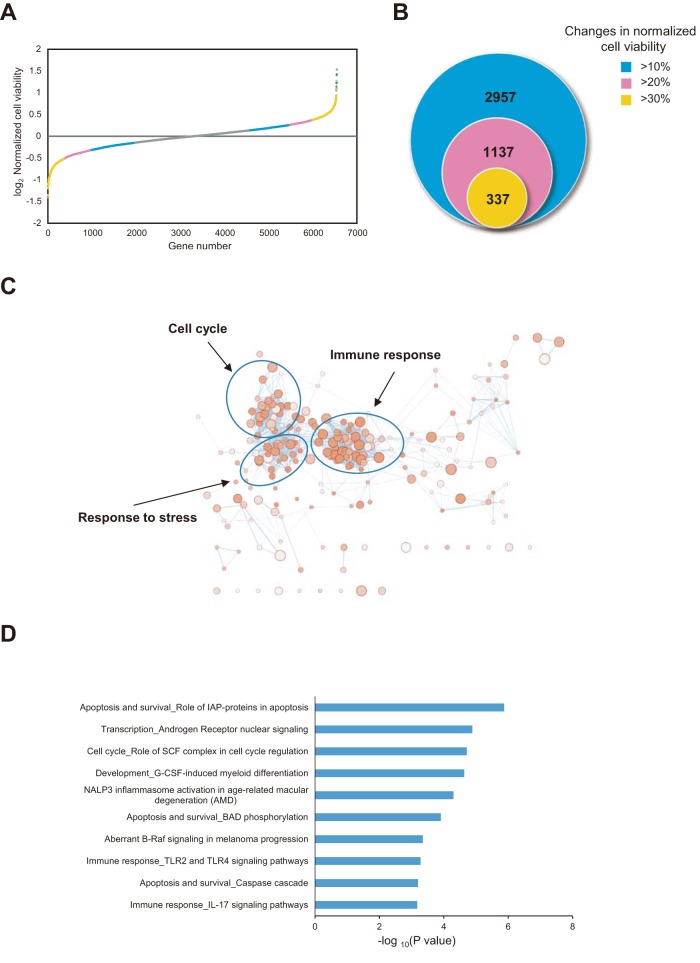
Genomewide RNAi screening to identify essential host factors for H5N1 infection. (A) Rank-ordered distribution of cell viability based on 6,540 human genes in the second round of genomewide RNAi screening. (B) Counts of genes associated with changes in cell viability of more than 10%, 20%, or 30% in two genomewide RNAi screens. (C) GO term (biological process) enrichment of 337 genes based on Metacore analysis. Enrichment was visualized using the Enrichment Map application in Cytoscape. Biological process terms are presented as nodes, while the node color indicates the significance of terms and node size reflects the object count enriched in the terms. The connection between terms is based on shared objects. (D) Functional enrichment of the top 10 significant pathways for 337 genes (false discovery rate < 0.05). IAP, inhibitor of apoptosis; SCF, Skp, Cullin, F-box-containing complex; G-CSF, granulocyte colony-stimulating factor; NALP3, NACHT, LRR, and PYD domain-containing protein 3; BAD, BCL2-associated agonist of cell death; TLR, Toll-like receptor; IL-17, interleukin-17.

A total of 19,424 human genes were screened by culturing with RNAi; 6,540 effective genes were selected for a second round of genomewide RNAi screening. The viability of H5N1-infected A549 cells was affected by a total of 2,957 genes (Fig. 1A and B; see also Fig. S1 and Table S1 in the supplemental material). Among these host genes, 1,137 and 337 RNAi treatments altered cell viability with H5N1 infection by more than 20% and 30%, respectively, in both screenings (Fig. 1B). The Metacore database indicated that the most significant Gene Ontology (GO) terms for biological process clusters for the 337 genes correspond to immune responses, the cell cycle, and stress responses (Fig. 1C). On the basis of enrichment of the GO term cellular component (CC) as well as on protein-protein interaction (PPI) analysis using the STRING database (https://string-db.org/), knockdown of the genes related to spliceosomes ameliorated cell death; in contrast, knockdown of the genes related to mitochondrial respiratory chain complex I aggravated cell death (Fig. S2). Among the top 10 functional enrichment pathways, 4 were related to the immune response and 3 to apoptosis (Fig. 1D). Our results obtained through the RNAi platform show that cell viability may be influenced by over a thousand host genes and that those genes might represent potential drug targets against H5N1 virus.

10.1128/mSystems.00431-19.1 FIG S1 Genomewide RNAi screen for host genes associated with H5N1 virus infection. (A) Flow chart of the genomewide RNAi screen of 19,424 genes to identify those important in influenza A H5N1 virus infection. (B) Heat map of cell viability related to 337 genes whose knockdown altered cell viability by >30% in each of two siRNA screens. The two columns of the heat map represent the first and second rounds of genomewide RNAi screening. Download FIG S1, PDF file, 0.4 MB. Copyright © 2019 Zhang et al. 2019 Zhang et al. This content is distributed under the terms of the Creative Commons Attribution 4.0 International license.

10.1128/mSystems.00431-19.2 FIG S2 Functional enrichment of essential host genes associated with H5N1 infection. (A) Enriched Gene Ontology cellular component terms associated with 337 genes, as analyzed using Metacore. (B) Interactions among clusters of spliceosomes. The three kinds of colored circles (dark red, medium red, and light red) indicate that the cell viability increased more than 30%, 20%, and 10%, respectively. (C) Interactions among the clusters of mitochondrial respiratory chain complex I. The three kinds of colored circles (dark blue, medium blue, and light blue) indicate that the cell viability decreased more than 30%, 20%, and 10%, respectively. Download FIG S2, PDF file, 1.4 MB. Copyright © 2019 Zhang et al. 2019 Zhang et al. This content is distributed under the terms of the Creative Commons Attribution 4.0 International license.

10.1128/mSystems.00431-19.7 TABLE S1 Host genes identified in the genomewide RNAi screen. Download Table S1, XLSX file, 0.3 MB. Copyright © 2019 Zhang et al. 2019 Zhang et al. This content is distributed under the terms of the Creative Commons Attribution 4.0 International license.

### Selection of candidate drugs and screening in H5N1-infected A549 cells.

**FIG 2:**
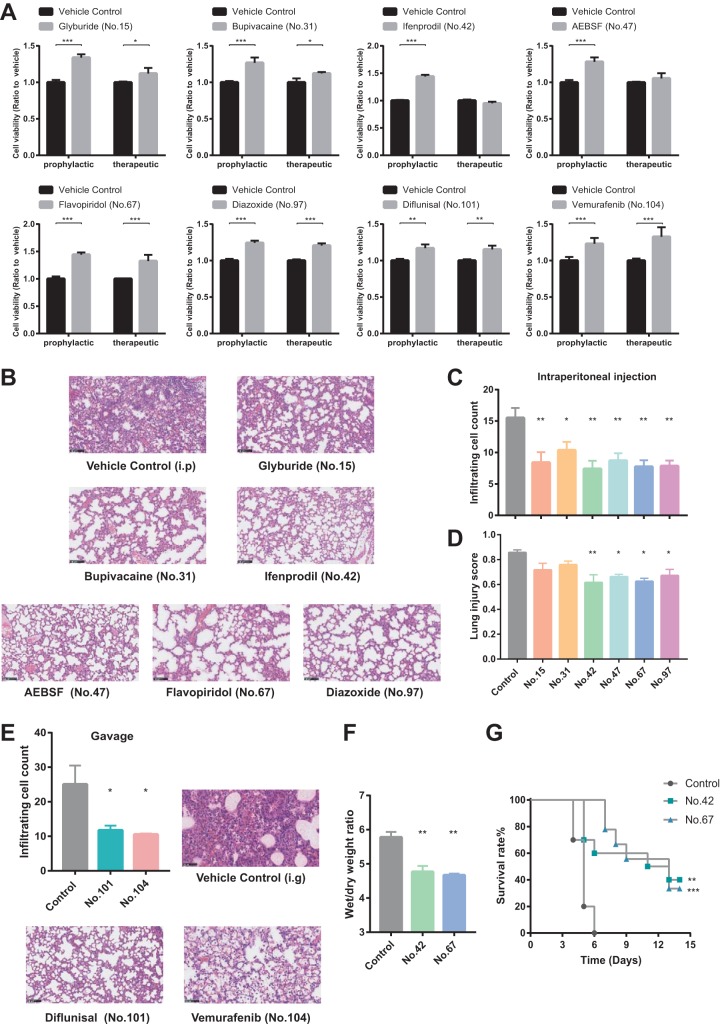
Drugs effective against influenza A H5N1 virus infection *in vitro* and *in vivo.* (A) Viability of A549 cells infected with H5N1 at a multiplicity of infection of 3.0 and treated with drugs prophylactically (3 h before infection) or therapeutically (3 h after infection). Viability was determined at 48 h after infection and is expressed relative to vehicle-treated control cells. (B) Representative images of lung pathology in hematoxylin-and-eosin-stained sections from H5N1-infected C57BL/6 mice (*n* = 3 to 5) treated with drugs or vehicle by intraperitoneal injection. Scale bar = 100 μm. Lung tissues were removed at 3 days after infection. i.p, intraperitoneal. (C and D) Numbers of infiltrating cells (C) and lung injury scores (D) per microscopic field (means ± standard errors of the means [SEM]) are shown in the bar graph (*n* = 100 fields per treatment). (E) Representative images of lung pathology in hematoxylin-and-eosin-stained sections from H5N1-infected C57BL/6 mice (*n* = 3 to 5) treated with drugs or vehicle by intragastric administration. Lung tissues were removed at 3 days after infection. The numbers of infiltrating cells per microscopic field (means ± SEM) are shown in the bar graph (*n* = 100 fields per treatment). Scale bar = 100 μm. i.g, intragastric. (F) Wet-to-dry-weight ratios of lung tissue from H5N1-infected C57BL/6 mice treated by intraperitoneal injection with ifenprodil (no. 42), flavopiridol (no. 67), or vehicle. *In vitro* and *in vivo* experiments were repeated three times. *, *P* < 0.05; **, *P* < 0.01; ***, *P* < 0.001 (two-tailed *t* test). (G) Kaplan-Meier survival curves of H5N1-infected C57BL/6 mice treated with ifenprodil (no. 42), flavopiridol (no. 67), or vehicle by intraperitoneal injection. **, *P* < 0.01; ***, *P* < 0.001 (log rank test). Detailed information about effective drug concentrations *in vitro* and drug dosages *in vivo* are shown in Table S3.

By searching the DrugBank database, we identified 146 drug target genes (372 drug candidates) among 1,137 host genes that altered cell viability by more than 20% in the RNAi screen after H5N1 virus infection. We further evaluated the prophylactic and therapeutic effects of 104 commercially available drugs (with 65 validated target genes) by examining their ameliorative effects in a cell viability assay in H5N1-infected A549 cells (Fig. S3A and B and Table S2) and found 28 drugs that significantly enhanced cell viability with low cytotoxicity (Fig. 2A and Fig. S3C; Table S3).

10.1128/mSystems.00431-19.3 FIG S3 *In vitro* validation of drugs against H5N1 virus infection. (A) Flow chart of drug screening based on 1,137 genes and 104 drug candidates. (B) Flow chart of the *in vitro* validation of drug efficacy against H5N1 infection. (C) Viability of A549 cells infected with H5N1 at a multiplicity of infection of 3.0 and treated with drugs prophylactically (3 h before infection) or therapeutically (3 h after infection). Viability was assessed at 48 h after infection and is expressed relative to that of vehicle-treated control cells. *In vitro* experiments were repeated three times. *, *P* < 0.05; **, *P* < 0.01; ***, *P* < 0.001 (two-tailed *t* test). Download FIG S3, PDF file, 0.3 MB. Copyright © 2019 Zhang et al. 2019 Zhang et al. This content is distributed under the terms of the Creative Commons Attribution 4.0 International license.

10.1128/mSystems.00431-19.8 TABLE S2 Drug information. Download Table S2, XLSX file, 0.01 MB. Copyright © 2019 Zhang et al. 2019 Zhang et al. This content is distributed under the terms of the Creative Commons Attribution 4.0 International license.

10.1128/mSystems.00431-19.9 TABLE S3 *In vitro* and *in vivo* drug information. Download Table S3, XLSX file, 0.01 MB. Copyright © 2019 Zhang et al. 2019 Zhang et al. This content is distributed under the terms of the Creative Commons Attribution 4.0 International license.

### Drug candidates that ameliorated ALI and improved survival rates in H5N1-infected mice.

Among the 28 drugs with which we observed a significant recovery of cell viability following H5N1 infection, 8 were glucocorticoids, which, at low doses, are used in conventional treatments against influenza virus-induced ALI (18, 19). Therefore, we focused on the other 20 nonhormonal drug candidates and tested the top 10 nonhormonal drug candidates that enhanced the viability of H5N1-infected A549 cells against ALI in C57BL/6 mice infected with H5N1 (Fig. S4A; see also Table S3). The following 8 of those 10 drugs significantly decreased the number of infiltrating cells in mouse lung tissues: glyburide (no. 15), bupivacaine (no. 31), ifenprodil (no. 42), 4-(2-aminoethyl) benzenesulfonyl fluoride (AEBSF) (no. 47), flavopiridol (no. 67), diazoxide (no. 97), diflunisal (no. 101), and vemurafenib (no. 104) (Fig. 2B, C, and E). The following 4 of those 8 drugs significantly reduced lung injury scores in animals: ifenprodil, AEBSF, flavopiridol, and diazoxide (Fig. 2B and D; see also Fig. S4B). Both ifenprodil treatment and flavopiridol treatment ameliorated lung edema by decreasing the wet-to-dry-weight ratio in H5N1-infected mice with induced ALI (Fig. 2F; see also Fig. S4C and D). We measured the impact of these 2 drugs on major subsets of leukocytes during the response to H5N1 virus infection and found that ifenprodil or flavopiridol significantly decreased the levels of neutrophils, natural killer (NK) cells, and T cells in H5N1 virus-infected mouse lung tissues (Fig. S5). Notably, both ifenprodil treatment and flavopiridol treatment significantly increased the survival rate of H5N1 virus-infected mice (Fig. 2G). The weight loss of mice administered ifenprodil or flavopiridol was recovered at the second week after H5N1 virus infection (Fig. S4E). Taken together, these results indicate that 8 of the top 10 nonhormonal drugs can ameliorate ALI in H5N1-infected mice, with ifenprodil and flavopiridol being the most effective.

10.1128/mSystems.00431-19.4 FIG S4 *In vivo* validation of drugs against H5N1 infection. (A) Flow chart of the *in vivo* validation of drug efficacy. (B) Lung injury scores of tissue from H5N1-infected C57BL/6 mice (*n* = 3 to 5) treated with drug candidates by intragastric administration. Lung tissues were obtained at 3 days after infection. Lung injury scores per microscopic field are shown in the bar graph. *n* = 100 fields were analyzed for each group. (C and D) Wet-to-dry-weight ratios of lung tissue from H5N1-infected C57BL/6 mice treated with drugs by intraperitoneal injection (C) or intragastric administration (D). Lung tissues were obtained at 3 days after infection. Compound identities: no. 15, glyburide; no. 31, bupivacaine; no. 47, AEBSF; no. 97, diazoxide; no. 101, diflunisal; no. 104, vemurafenib. The data shown represent means ± standard errors of the means (SEMs). *In vivo* experiments were repeated three times. *, *P* < 0.05; **, *P* < 0.01 (two-tailed test). (E) Body weight changes in H5N1-infected C57BL/6 mice treated with ifenprodil (no. 42), flavopiridol (no. 67), or vehicle by intraperitoneal injection. Download FIG S4, PDF file, 1.4 MB. Copyright © 2019 Zhang et al. 2019 Zhang et al. This content is distributed under the terms of the Creative Commons Attribution 4.0 International license.

10.1128/mSystems.00431-19.5 FIG S5 Ifenprodil and flavopiridol impact inflammatory cell infiltration. (A) Representative images of lung pathology by immunofluorescence staining from H5N1-infected C57BL/6 mice (*n* = 3 to 5) treated with ifenprodil (no. 42), flavopiridol (no. 67), or vehicle by intraperitoneal injection. Lung tissues were removed at 3 days after infection. The blue areas indicate cell nuclei stained with DAPI, the red areas indicate CD45 protein (leukocyte common antigen), and the green areas indicate marker proteins. Scale bar, 100 μm. The numbers of neutrophil cells (CD45^+^ MPO^+^), T cells (CD45^+^ CD3e^+^), and NK cells (CD45^+^ NCR1^+^) per microscopic field are shown in the bar graph (means ± SEM, *n* = 100 fields per treatment). (B and C) The numbers of macrophage cells (B) and dendritic cells (C) per microscopic field are shown in the bar graph (means ± SEM, *n* = 100 fields per treatment). *, *P* < 0.05; **, *P* < 0.01 (two-tailed *t* test). Download FIG S5, PDF file, 2.2 MB. Copyright © 2019 Zhang et al. 2019 Zhang et al. This content is distributed under the terms of the Creative Commons Attribution 4.0 International license.

### Ifenprodil and flavopiridol attenuated H5N1-induced cytokine storm in mice.

**FIG 3:**
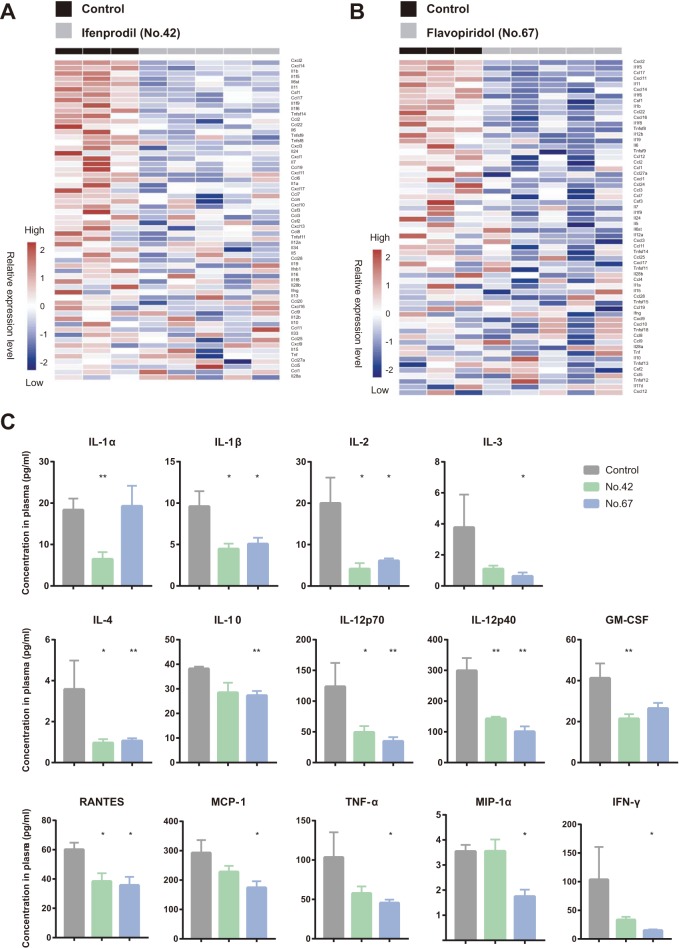
Ifenprodil and flavopiridol alleviated H5N1-induced cytokine storm in mouse lungs. (A and B) Heat maps of relative cytokine mRNA levels in lung tissues of H5N1-infected C57BL/6 mice treated with ifenprodil (no. 42) (A), flavopiridol (no. 67) (B), or vehicle. Lung tissues were obtained at 3 days after infection, and total RNA was isolated for RNA sequencing. The columns of the heat maps represent each experimental mouse. The relative expression levels of cytokine mRNAs were normalized by Z-score transformation. (C) The levels of cytokines and chemokines in plasma from H5N1-infected C57BL/6 mice treated with ifenprodil (no. 42), flavopiridol (no. 67), or vehicle. Blood samples were obtained at 3 days after infection. *, *P* < 0.05; **, *P* < 0.01 (Mann-Whitney U test). GM-CSF, granulocyte-macrophage colony-stimulating factor; TNF-α, tumor necrosis factor alpha; IFN-γ, gamma interferon; RANTES, regulated on activation normally T-cell expressed and secreted; MCP-1, metaphase chromosome protein 1; MIP-1α, macrophage inflammatory protein 1 alpha.

RNA sequencing (RNA-Seq) analysis of the lung tissue showed that ifenprodil and flavopiridol alleviated cytokine storm in H5N1-infected mice (Fig. 3A and B; see also Fig. S6A and B). We obtained blood samples from H5N1-infected mice and performed multiplex analyses to measure the levels of 23 cytokines/chemokines in plasma from mice administered ifenprodil or flavopiridol. Overall, ifenprodil treatment and flavopiridol treatment significantly decreased the measured levels of 8 and 12 cytokines/chemokines, respectively, in the plasma of H5N1-infected mice (Fig. 3C). The other six drugs decreased the mRNA levels of several cytokines and cytokine receptors in mouse blood (Fig. S6C to H). Our results suggest that these drugs, especially ifenprodil and flavopiridol, can attenuate the excessive inflammation caused by H5N1 virus.

10.1128/mSystems.00431-19.6 FIG S6 Drugs antagonize H5N1-induced upregulation of cytokines. (A and B) Relative mRNA levels of cytokines and chemokines in H5N1-infected C57BL/6 mice (*n* = 3 to 5) treated with ifenprodil (no. 42) (A), flavopiridol (no. 67) (B), or vehicle. Lung tissue samples were collected at 72 h after infection, and total RNA was isolated for RNA sequencing. #, *P* < 0.1; *, *P* < 0.05; **, *P* < 0.01 (two-tailed *t* test). (C to H) Relative mRNA levels of cytokines and cytokine receptors in H5N1-infected C57BL/6 mice (*n* = 3 to 5) treated with glyburide (no. 15) (C), bupivacaine (no. 31) (D), AEBSF (no. 47) (E), diazoxide (no. 97) (F), diflunisal (no. 101) (G), vemurafenib (no. 104) (H), or vehicle. Blood samples were collected at 72 h after infection, and total RNA was isolated for RNA sequencing. #, *P* < 0.1; *, *P* < 0.05; **, *P* < 0.01 (two-tailed *t* test). Download FIG S6, PDF file, 0.8 MB. Copyright © 2019 Zhang et al. 2019 Zhang et al. This content is distributed under the terms of the Creative Commons Attribution 4.0 International license.

### Ifenprodil-targeted NMDA receptor genes and flavopiridol-targeted cyclin-dependent kinase 4 (CDK4) genes are linked to lung injury.

**FIG 4:**
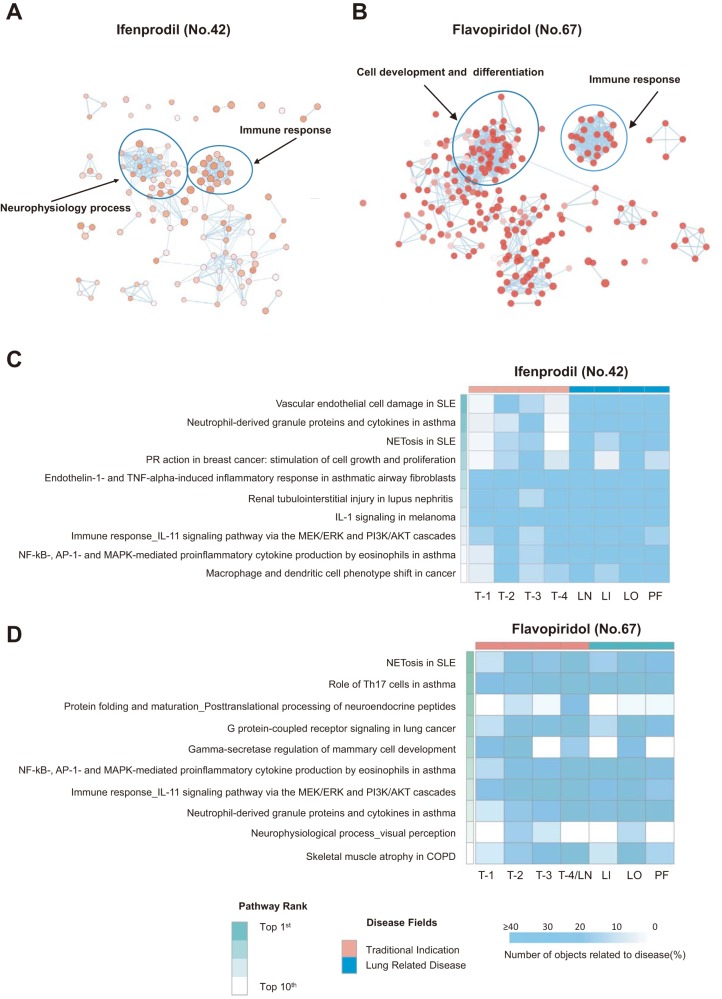
Functional processes and pathways influenced by ifenprodil and flavopiridol in H5N1-infected mice. (A and B) GO term (biological process) enrichment of DEGs associated with ifenprodil (no. 42) treatment (A) or flavopiridol (no. 67) treatment (B) in H5N1-infected mice, as analyzed by Metacore. Enrichment was visualized using the Enrichment Map application in Cytoscape. Biological processes are presented as nodes, while the node color indicates the significance of the biological processes, and the node size reflects the object count enriched in the biological processes. The connection between biological processes is based on shared objects. (C and D) Heat maps of the percentage of objects related to the traditional indication or to lung diseases in the top 10 enriched pathways associated with ifenprodil (no. 42) treatment (C) or flavopiridol (no. 67) treatment (D). LN, lung neoplasms; LI, lung diseases (interstitial); LO, lung diseases (obstructive); PF, pulmonary fibrosis; T, traditional indication; SLE, systemic lupus erythematosus; NETosis, neutrophil extracellular trap-associated cell death; PR, progesterone receptor; ERK, extracellular signal-regulated kinase; PI3K, phosphatidylinositol 3-kinase; MAPK, mitogen-activated protein kinase; COPD, chronic obstructive pulmonary disease. Detailed information about the enriched pathways and objects is provided in Table S4.

Ifenprodil is a selective N-methyl-d-aspartate (NMDA) receptor antagonist (20) and has been clinically used to treat neuronal injury and neurological disorders induced by overstimulation of the NMDA receptor (21). Our RNAi screening data revealed that knockdown of the NMDA receptor gene (GRIN2B) could alter the viability of H5N1-infected cells (Table S1). We obtained RNA sequencing data from lung tissues of H5N1-infected mice treated with ifenprodil and analyzed biological processes and pathways influenced by ifenprodil compared to vehicle control mice. We found that ifenprodil influenced the immune response and neurophysiological processes in the mouse lung (Fig. 4A). Nine of the top 10 pathways of the ifenprodil treatment group were linked to the immune response (Fig. 4C). Dozens of genes in most of the top 10 pathways were reported to be related to lung diseases as well as to traditional neuropathic indications of ifenprodil (Fig. 4C; see also Table S4 in the supplemental material). Although previous reports indicated that NMDA receptors are expressed in lung tissues, that NMDA receptor signaling is linked to inflammation, and that overstimulation of the NMDA receptor can trigger lung injury (22, 23), our study revealed for the first time that ifenprodil is effective in avian influenza A H5N1 virus infection and lung injury.

10.1128/mSystems.00431-19.10 TABLE S4 Traditional/lung disease-related objects in the top 10 pathways for ifenprodil (no. 42) and flavopiridol (no. 67) administration. Download Table S4, XLSX file, 0.06 MB. Copyright © 2019 Zhang et al. 2019 Zhang et al. This content is distributed under the terms of the Creative Commons Attribution 4.0 International license.

Flavopiridol is an inhibitor of cyclin-dependent kinases (CDKs) (including CDK1, CDK2, CDK4, CDK5, CDK6, CDK7, CDK8, and CDK9) and has been investigated for the treatment of several types of cancers, including leukemia, liver cancer, and renal cancer, in clinical phase 2 trials (24–26). Previous research found that CDK4 inhibition resulted in the improvement of lung injury by blocking leukocyte adhesion and migration and that flavopiridol inhibited avian influenza H7N9 virus replication in A549 cells but that its efficiency *in vivo* could not be confirmed (27). Our RNAi screening results showed that the viability of H5N1 virus-infected cells was increased due to CDK4 gene knockdown (Table S1). Using RNA sequencing data for biological process and pathway analysis, we found that flavopiridol treatment could alter the immune response as well as cell development and differentiation in H5N1 virus-infected mouse lung tissues (Fig. 4B). Among the top 10 pathways of the flavopiridol treatment group, 5 are related to the immune response of lung disease and 2 are involved in cancer (Fig. 4D). We found that dozens of genes in most of the top 10 pathways were linked to lung diseases as well as to the known antineoplastic function of flavopiridol (Fig. 4D; see also Table S4). Our study revealed for the first time that flavopiridol is effective in lung injury.

## DISCUSSION

In this study, we established an efficient whole-genome RNAi-based platform for repurposing drugs for H5N1 infection in A549 cells. Compared to primary epithelial cells, A549 cells are widely used in large-scale experiments for the study of influenza A virus based on convenience and economic considerations (11, 13, 15). We identified potential gene targets via a genomewide RNAi screen, in which we searched for drugs that would maximize the viability of H5N1-infected cells. Most genomewide RNAi screens use replication of the moderately pathogenic influenza H1N1 virus as the readout of interference (8–15). Since the highly pathogenic avian influenza H5N1 virus can cause significant cell death (28) and since cell viability is highly correlated to virus lethality, we designed our genomewide RNAi screening platform to use cell death as the readout in order to identify repurposable drugs for treatment of H5N1 virus infection. Of the 28 effective drugs identified *in vitro*, 8 were glucocorticoids, which are already used to treat ALI in patients (18, 19). We showed that 8 of the top 10 nonhormone drugs effectively ameliorated ALI in H5N1-infected mice. We identified the neurological drug ifenprodil and the anticancer drug flavopiridol as potential novel effective remedies for avian influenza A H5N1 virus-infected lung injury.

The mechanisms by which ifenprodil and flavopiridol ameliorate lung injury and improve the survival of H5N1-infected mice may operate via an influence on the host immune response. Previous studies reported that limiting the infiltration of leukocytes such as NK cells and neutrophils could alleviate influenza A virus-induced ALI (29, 30). We found that both ifenprodil and flavopiridol significantly decreased the number of infiltrating cells in H5N1-infected mouse lung tissues. In addition, hypercytokinemia was reported in the blood samples of patients with lethal avian influenza A virus infection, and plasma cytokine and chemokine levels were linked to disease fatal outcomes (31, 32). In our study, both ifenprodil and flavopiridol alleviated cytokine storm, as they significantly decreased the RNA and protein levels of some cytokines and chemokines in lung tissues and blood samples from mice with H5N1 virus infection. In addition, we obtained H5N1-infected mouse lung tissues for RNA sequencing and conducted bioinformatic analysis, which showed that the genes influenced by ifenprodil or flavopiridol administration were highly clustered in GO functional terms and pathways related to the immune response. From these collective results, we speculated that the impact of ifenprodil and flavopiridol on the immune response contributes to the amelioration of H5N1-induced ALI and to improvement in mouse survival. However, the efficacy of ifenprodil and flavopiridol for the treatment of H5N1-induced ALI also needs clinical evaluation.

In our study, we evaluated the efficiency in H5N1-infected A549 cells of only 104 commercially available drugs from 372 drug candidates identified using our genomewide RNAi screening method; the efficiencies of the rest of the 268 drugs need to be further tested. Furthermore, we examined the efficiencies of only 10 of the 28 *in vitro*-effective drugs in an animal model; the rest of the drug candidates also need to be tested *in vivo*.

Our report provides an economical, quick, and highly effective method (compared with traditional drug development strategies) for identifying novel remedies against avian influenza virus-induced lung injury. This approach could be generalized for identifying other contexts in which drugs can be repurposed.

## MATERIALS AND METHODS

### Cells and virus.

A549 human lung adenocarcinoma epithelial cells (ATCC, Rockville, MD, USA) were maintained in Ham’s F12 nutrient medium (HyClone, Logan, UT, USA) supplemented with 10% fetal bovine serum (Gibco, Grand Island, NY, USA), 100 U ml^−1^ penicillin, and 100 U ml^−1^ streptomycin. Influenza virus A/Jilin/9/2004 (H5N1) was propagated by inoculation into specific-pathogen-free embryonated fowl eggs (10 to 11 days old) via the allantoic route. All *in vivo* and *in vitro* experiments involving live virus were performed in biosafety level 3 facilities.

### Genomewide siRNA screen.

A genomewide small interfering RNA (siRNA) library targeting 19,424 human genes (with three siRNAs targeting each gene) and negative-control (NC) siRNAs was purchased from RiboBio (Guangzhou, China). A549 cells were plated into 96-well plates and transfected with siRNA (100 nM) using Lipofectamine RNAiMAX reagent (Invitrogen, CA, USA). At 24 h after transfection, A549 cells were infected with H5N1 influenza virus (multiplicity of infection [MOI], 3.0) or administered an equal volume of allantoic fluid (AF). At 48 h after infection, cell viability was measured using the 3-(4,5-dimethylthiazol-2-yl)-5-(3-carboxymethoxyphenyl)-2-(4-sulfophenyl)-2H (MTS) assay (catalog no. G3582, Promega, WI, USA). Cell viability values were normalized by calculating the ratio with respect to the viability of cells transfected with NC siRNA in the same plates as follows:

$$\text{normalized cell viability}=\frac{{\text{siRNA}}^{\text{H5N1}}/{\text{siRNA}}^{\text{AF}}}{{\text{NC}}^{\text{H5N1}}/{\text{NC}}^{\text{AF}}}$$

where siRNA^H5N1^ represents the H5N1-infected siRNA group, siRNA^AF^ represents the AF-treated siRNA group, NC^H5N1^ represents the H5N1-infected NC group, and NC^AF^ represents the AF-treated NC group. A two-tailed *P* value of <0.05 was considered statistically significant. Genes associated with normalized cell viabilities that were increased or decreased by >10% were taken into the second round of screening. Detailed information about the host genes identified in the genomewide RNAi screen is provided in Table S1 in the supplemental material.

### Gene enrichment and network analyses.

The top hits in the siRNA screen were functionally grouped according to Gene Ontology (GO) terms and functional pathways as clustered by Metacore (Clarivate Analytics) software. The criteria used to identify “disease-related genes” followed the standards of Metacore (Clarivate Analytics) software. Cytoscape (version 3.6.1) with the Enrichment Map application (version 3.1.0) was used for visualization. Interactions among the genes were assessed using the STRING database (https://string-db.org/).

### Drugs.

A total of 1,137 genes were searched as drug targets in the DrugBank database (www.DrugBank.ca/), and 104 drugs were ultimately selected as candidates. All drugs were purchased from Selleck Chemicals (Houston, TX, USA), and detailed information about the drugs is provided in Table S2.

### *In vitro* experiments.

Drugs were added at 1, 10, and 100 μM prophylactically (3 h before infection) or therapeutically (3 h after infection) to A549 cells infected with H5N1 influenza virus, and cell viabilities were measured by MTS assay at 48 h after infection. We performed two rounds of screens to confirm drug efficacy. Drug cytotoxicity was measured by assessing the viability of drug-treated A549 cells that were not infected with H5N1. Negative-control cultures were treated with dimethyl sulfoxide (DMSO). Drugs that significantly improved the viability of H5N1-infected A549 cells and that showed low cytotoxicity in the two screens were considered effective for treating H5N1 infection *in vitro*. Drug information and the effective concentrations are listed in Table S3.

### *In vivo* experiments.

Wild-type C57BL/6 mice (6 to 8 weeks old; catalog no. 5653791, RRID MGI:5653791) were purchased from Vital River (Beijing, China) and divided into two groups (5 mice per group) based on whether drug was administered by intraperitoneal (i.p.) injection or by gavage. A control group was administered vehicle. Mice were intratracheally instilled with live H5N1 virus (10^6^ 50% tissue culture infectious doses [TCID_50_]), and they received drug or vehicle at 4 time points: 24 h and 3 h before and 24 h and 48 h after infection. Drug administration methods and concentrations are listed in Table S3. Mice were sacrificed at 3 days after viral infection, and bilateral lungs were collected to assess lung injury and pulmonary edema (33). The animal experiments in this work were approved by the Ethics Committee of the Institute of Basic Medical Sciences, Chinese Academy of Medical Sciences (ACUC-A02-2017-014) and adhered to the Chinese National Guidelines for the Care of Laboratory Animals and the Institutional Animal Care.

### Immunofluorescence staining.

Lung tissue was fixed with 4% formaldehyde for at least 24 h and was then embedded in paraffin. Next, antigen retrieval was performed. For immunofluorescence staining, sections were blocked in 1% bovine serum albumin (BSA) and subsequently incubated overnight with the following primary antibodies in phosphate-buffered saline (PBS) at the indicated dilutions: anti-CD45 (rat; Abcam), 1:200; anti-CD11b (rabbit; Abclonal), 1:100; anti-ITGAX (CD11c) (rabbit; Abclonal), 1:100; anti-NCR1 (rabbit; Abclonal), 1:100; anti-CD3e (rabbit; Abclonal), 1:100; antimyeloperoxidase (anti-MPO) (rabbit; CST), 1:500. Secondary antibodies conjugated to Alexa Fluor 488, Cy3, or 4′,6-diamidino-2-phenylindole (DAPI) were used at a dilution of 1:200. Whole-section images were scanned with a Nikon (Tokyo, Japan) Eclipse C1 fluorescence microscope and Nikon DS-U3 imaging system to identify leukocyte subsets.

### Measurement of cytokines and chemokines.

Three groups of five C57BL/6 mice (6 to 8 weeks old) were used in this study. Mice were infected with live H5N1 virus (10^6^ TCID_50_) and administered vehicle, ifenprodil (20 mg/kg/body weight, intraperitoneally), or flavopiridol (5 mg/kg, intraperitoneally) at 4 time points: 24 h and 3 h before and 24 h and 48 h after infection. Mouse plasma samples were collected 72 h after infection. The levels of cytokines and chemokines in the plasma were measured with a Bio-Plex mouse cytokine 23-plex panel in a Bio-Plex protein array system (Bio-Rad Laboratories).

### Measurement of the survival rate and body weight changes.

Wild-type C57BL/6 mice were divided into three groups (9 to 10 mice per group). Mice were administered vehicle, ifenprodil (20 mg/kg, intraperitoneally), or flavopiridol (5 mg/kg, intraperitoneally) at 24 h and 3 h before and 24 h and 48 h after infection (10^6^ TCID_50_ of H5N1 virus). The survival and body weight changes of the mice in each group were assessed daily for 14 days. The log rank test was used to compare the Kaplan-Meier survival curves.

### RNA sequencing and data analysis.

Lung tissue and blood from mice treated with drug or vehicle were obtained at 3 days after H5N1 infection. Total RNA was isolated from lung tissue and blood using TRIzol (Invitrogen, CA, USA). High-throughput strand-specific RNA sequencing was performed using an Illumina HiSeq 2500 platform (Berry Genomics, Beijing, China). Following quality control of the RNA sequencing reads by FastQC (version 0.11.2), the reads were mapped to the mouse genome (version mmc10; http://hgdownload.cse.ucsc.edu/downloads.html) with Bowtie2 (version 2.1.0) and TopHat2 (version 2.0.11). We then assembled the transcription units to calculate the number of mapped fragments per kilobase per million (FPKM) and analyzed the differentially expressed genes (DEGs) between samples using Cufflinks (version 2.2.1), Cuffmerge (version 2.2.1), and Cuffdiff (version 2.2.1). The functional pathways of DEGs between animals administered drug or vehicle were analyzed using Metacore.

### Statistical analysis.

Differences between two groups were assessed for significance using unpaired *t* tests or the Mann-Whitney U test. Differences among three or more groups were assessed using analysis of variance (ANOVA). The log rank test was used for Kaplan-Meier survival analysis. Statistical analysis was performed using GraphPad Prism 7.0. Differences associated with a two-tailed *P* value of <0.05 and a false-discovery-rate value (*q* value) of <0.05 were considered statistically significant.
