# Supplementary figures and images for: Ifenprodil and Flavopiridol Identified by Genomewide RNA Interference Screening as Effective Drugs To Ameliorate Murine Acute Lung Injury after Influenza A H5N1 Virus Infection
Source: mSystems. 2019 Dec 10;4(6):e00431-19. doi: 10.1128/mSystems.00431-19 (PMC6906739; doi:10.1128/mSystems.00431-19)

**A**

## SECOND SCREEN

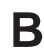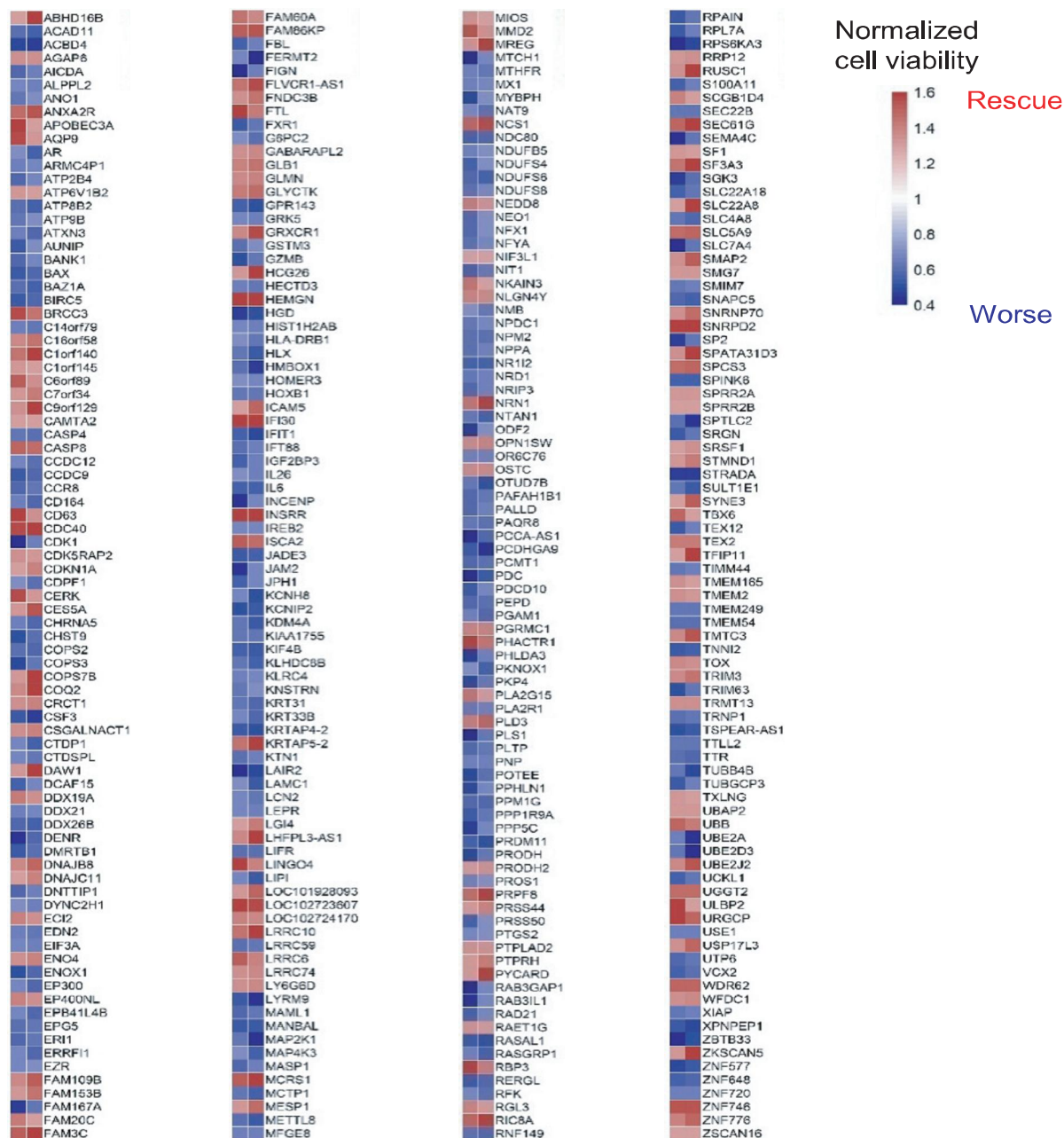

Supplement: FIG S1 [file mSystems.00431-19-sf001.pdf]

Figure S2

A

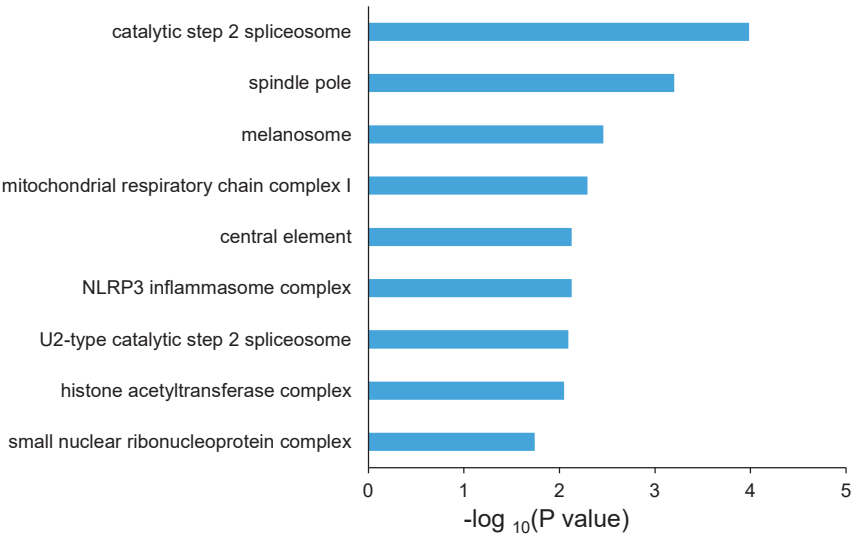

B

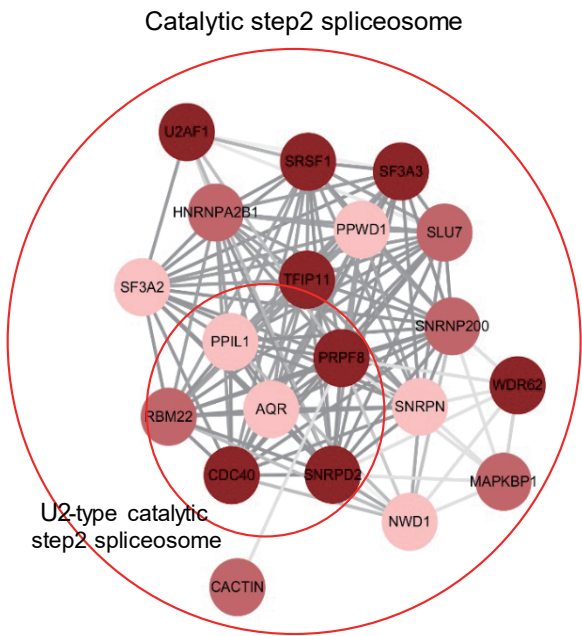

C

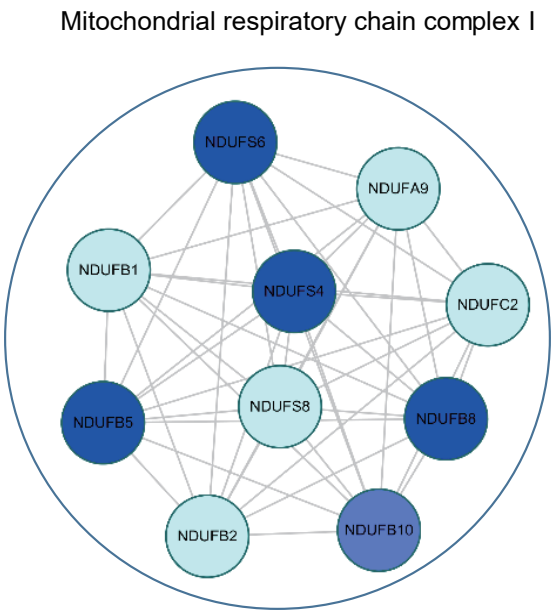

Supplement: FIG S2 [file mSystems.00431-19-sf002.pdf]

Figure S3

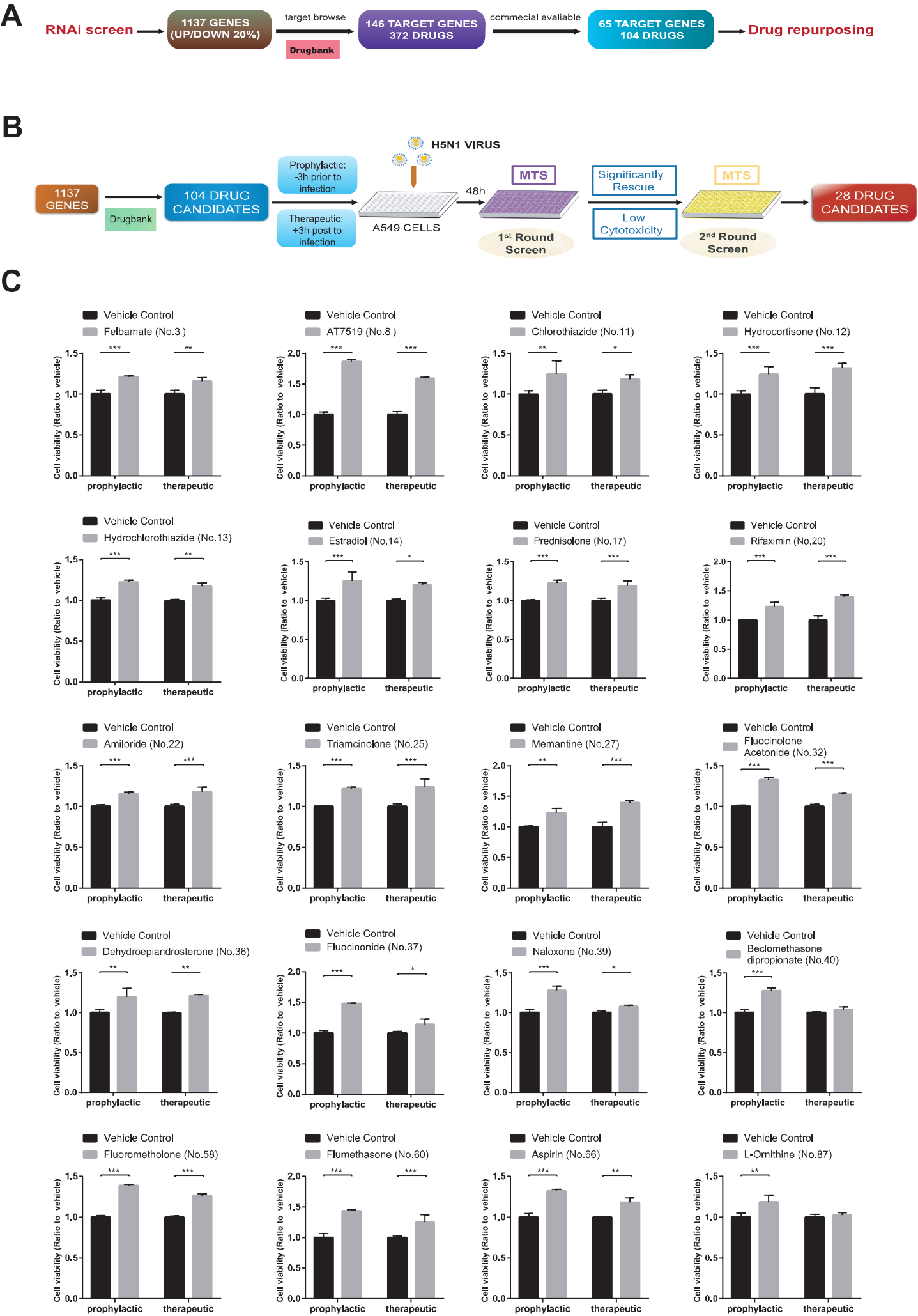

Supplement: FIG S3 [file mSystems.00431-19-sf003.pdf]

Figure S4

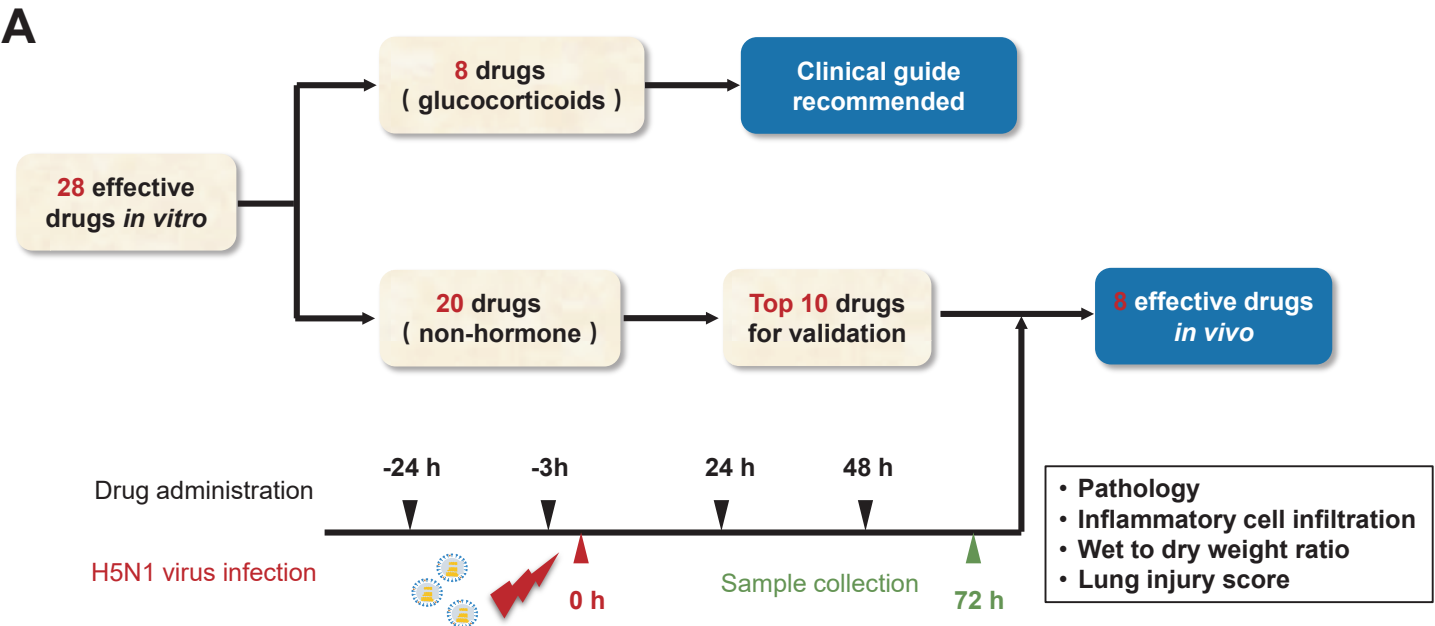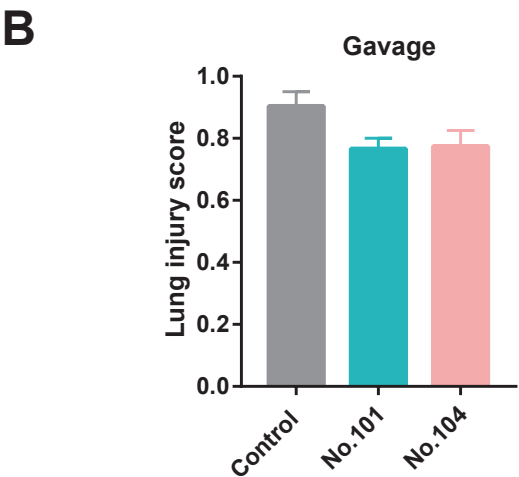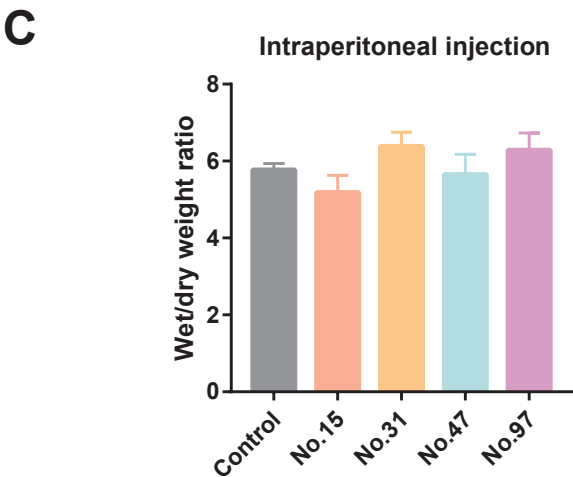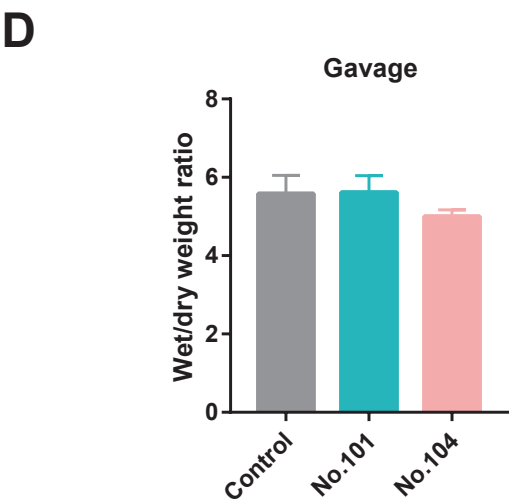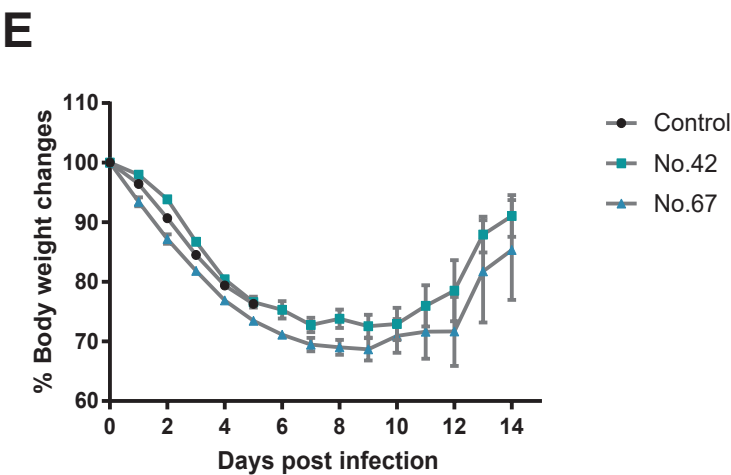

Supplement: FIG S4 [file mSystems.00431-19-sf004.pdf]

Figure S5

A

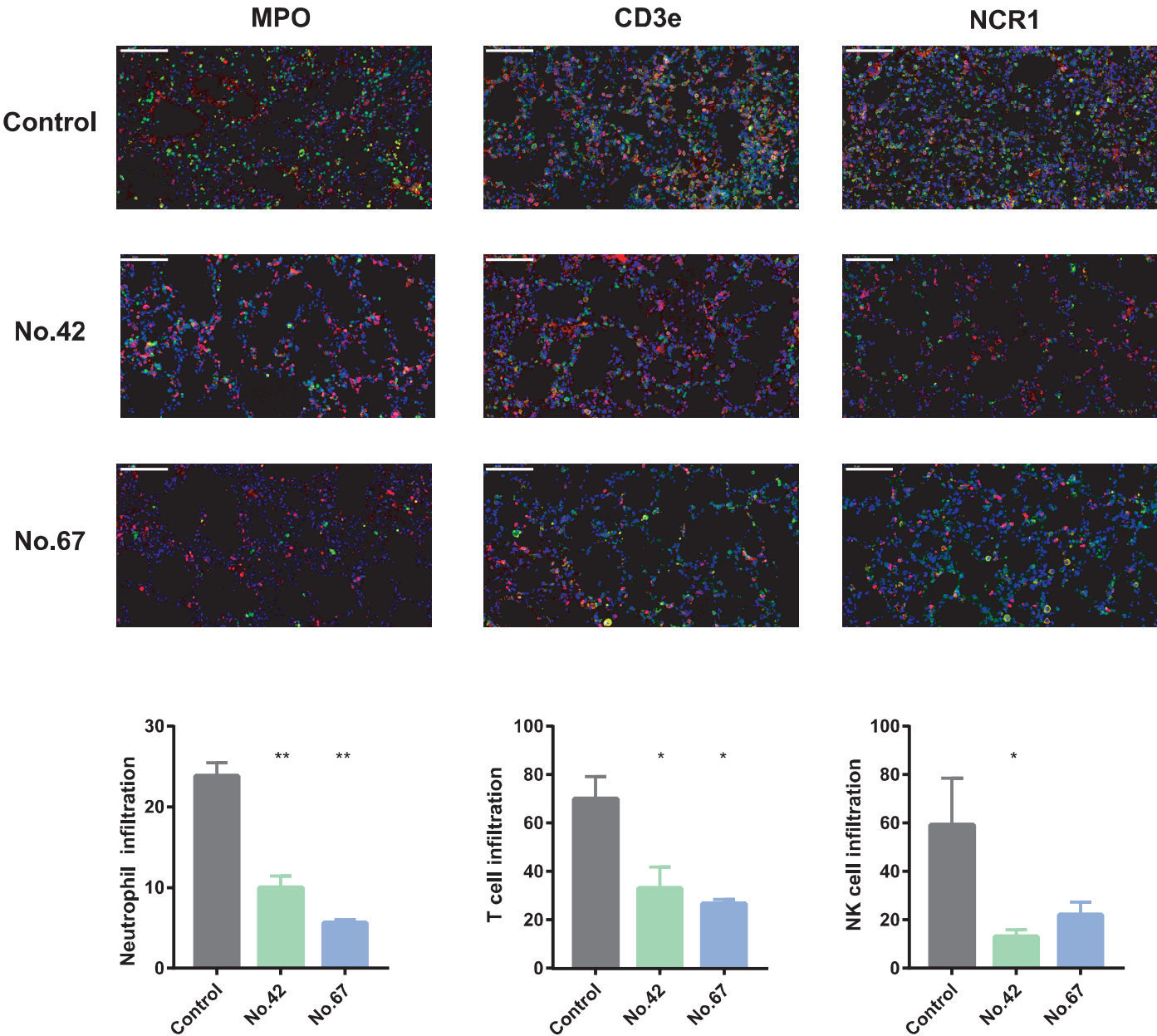

B

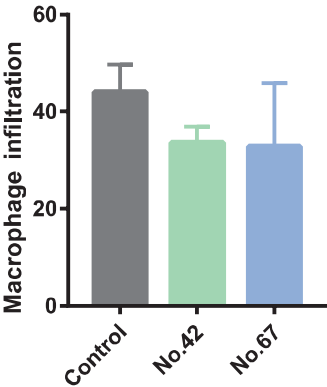

C

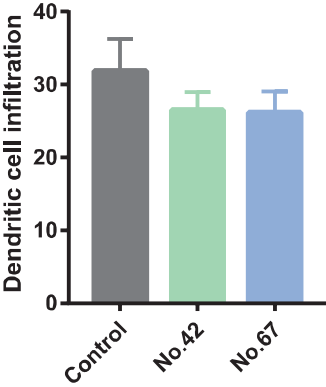

Supplement: FIG S5 [file mSystems.00431-19-sf005.pdf]

Figure S6

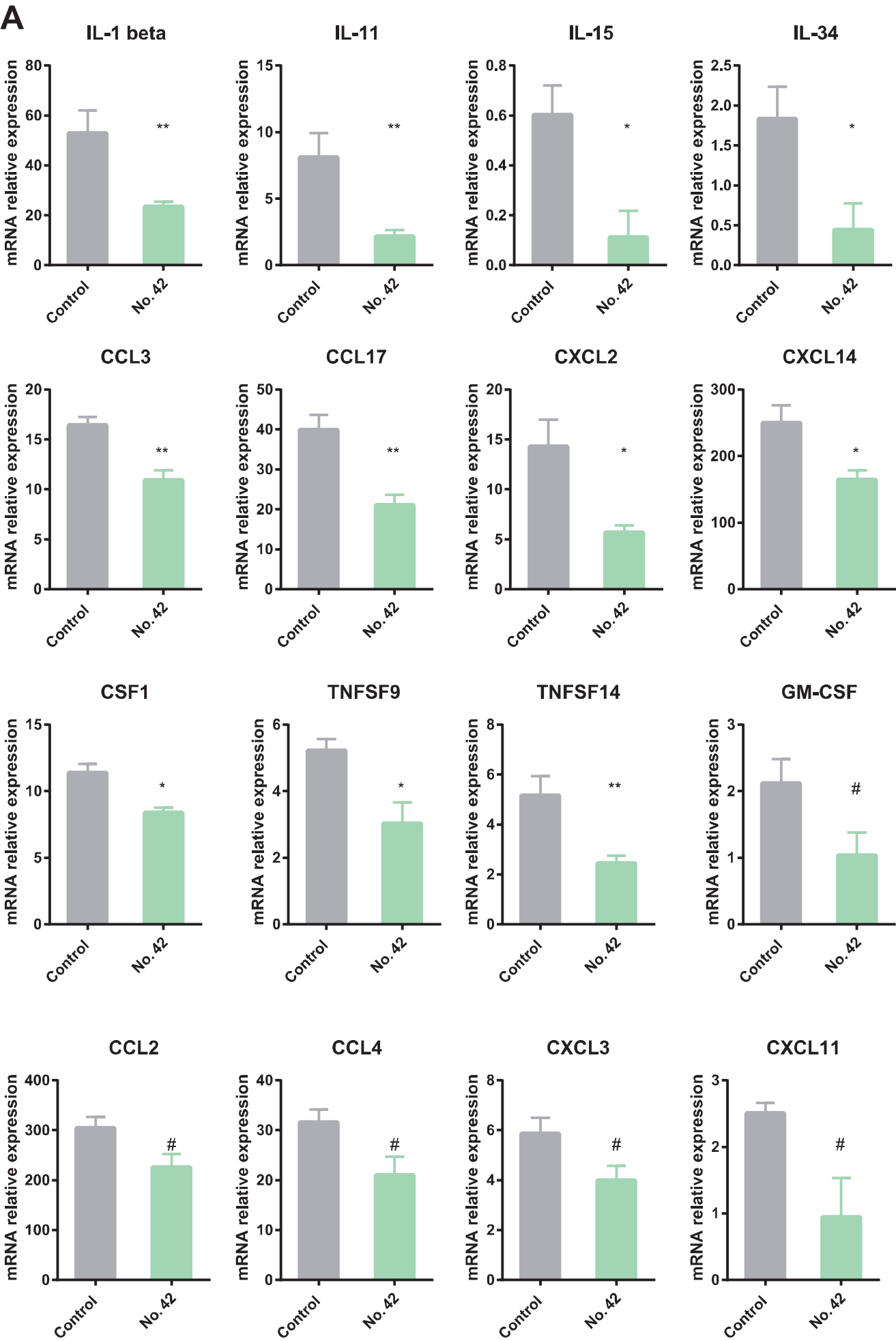

**B**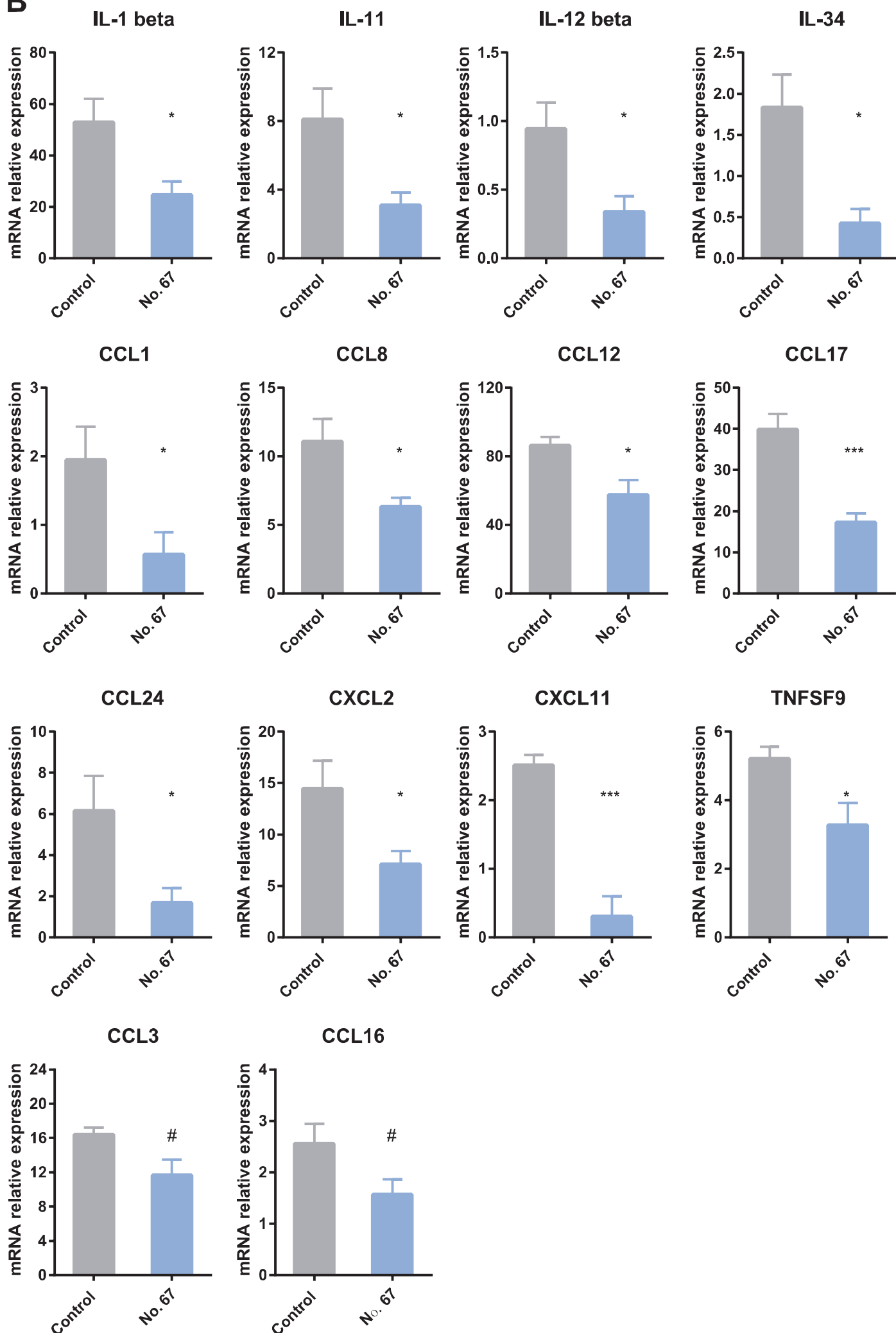

**C**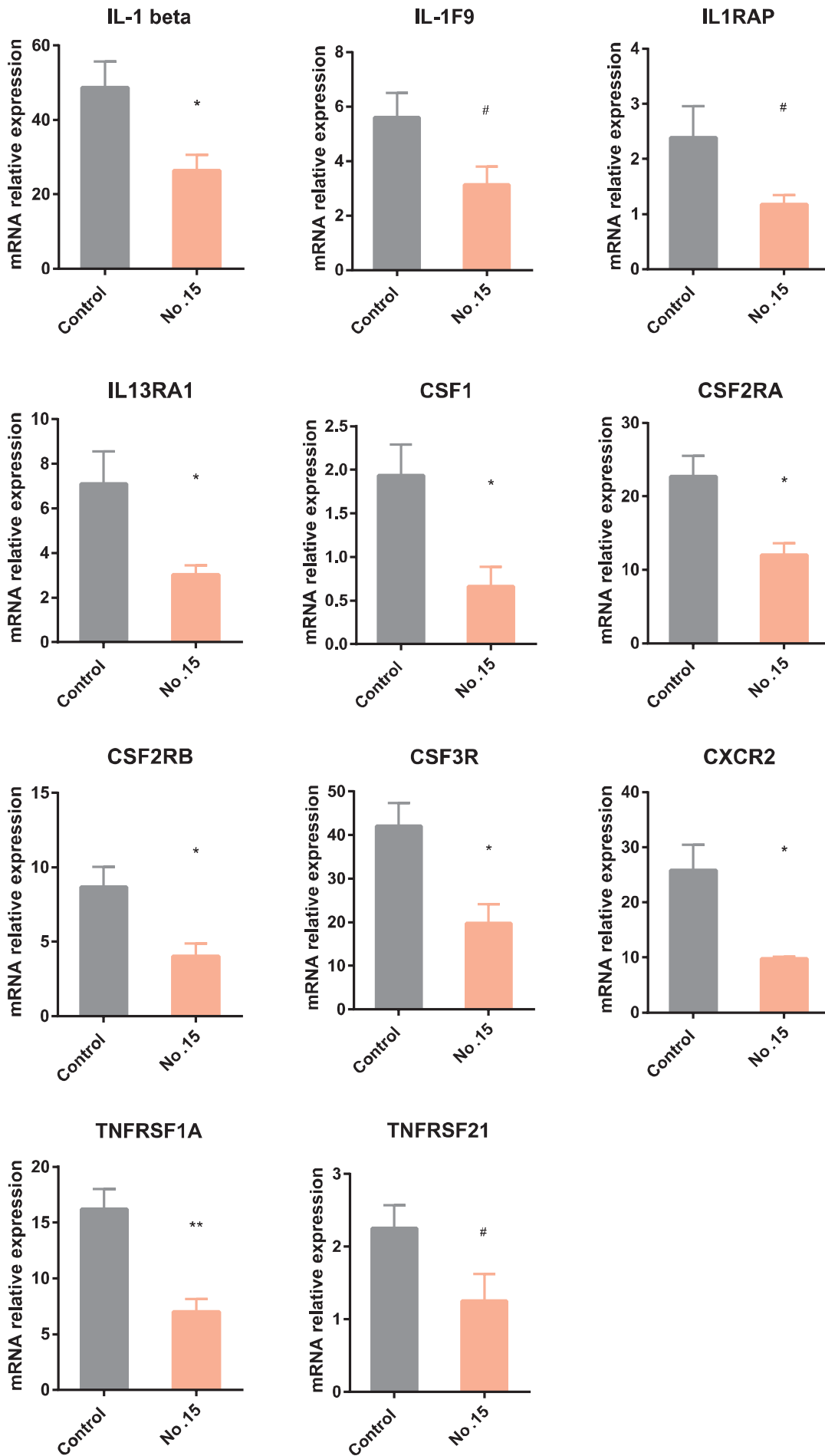

**D**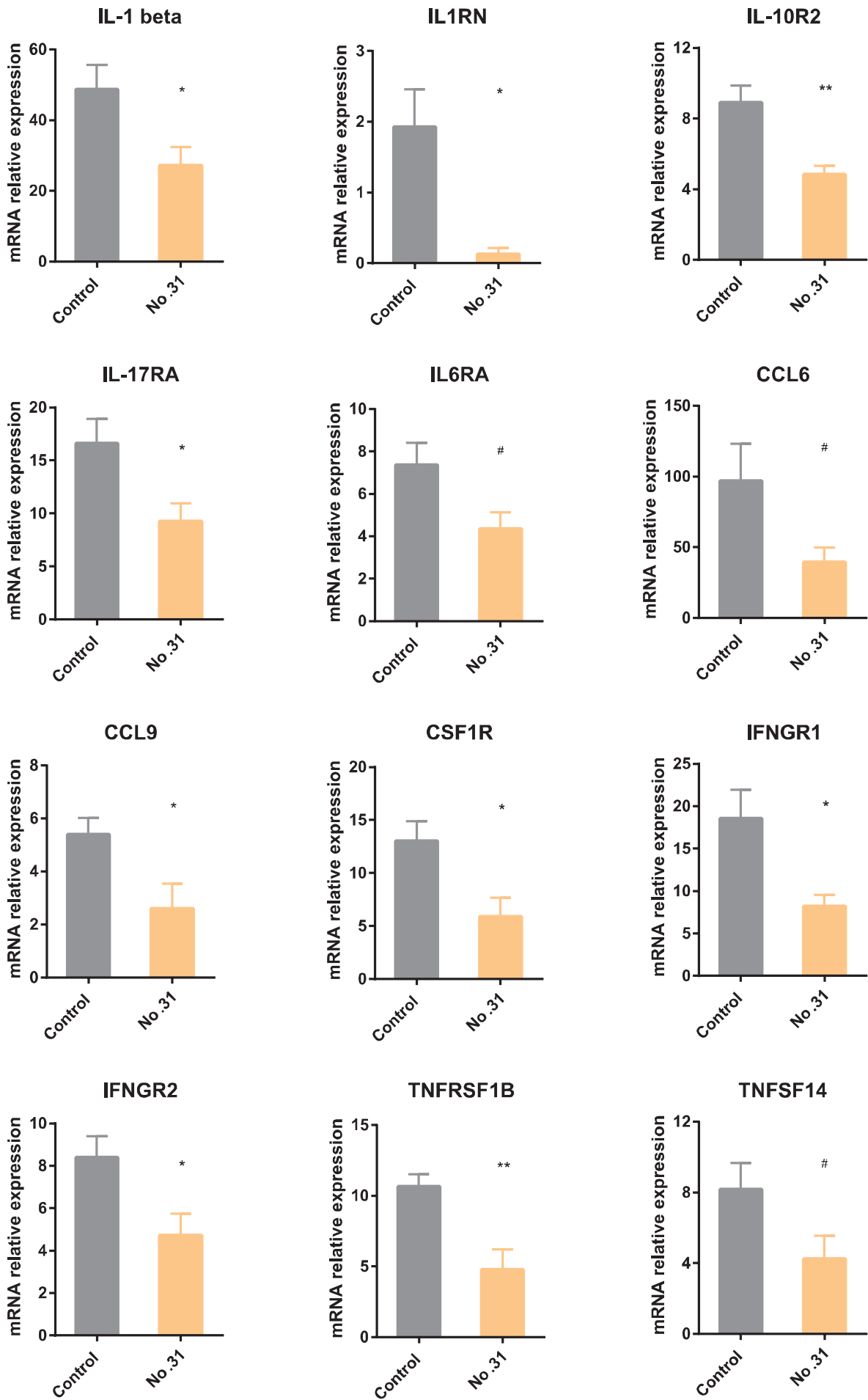

**E**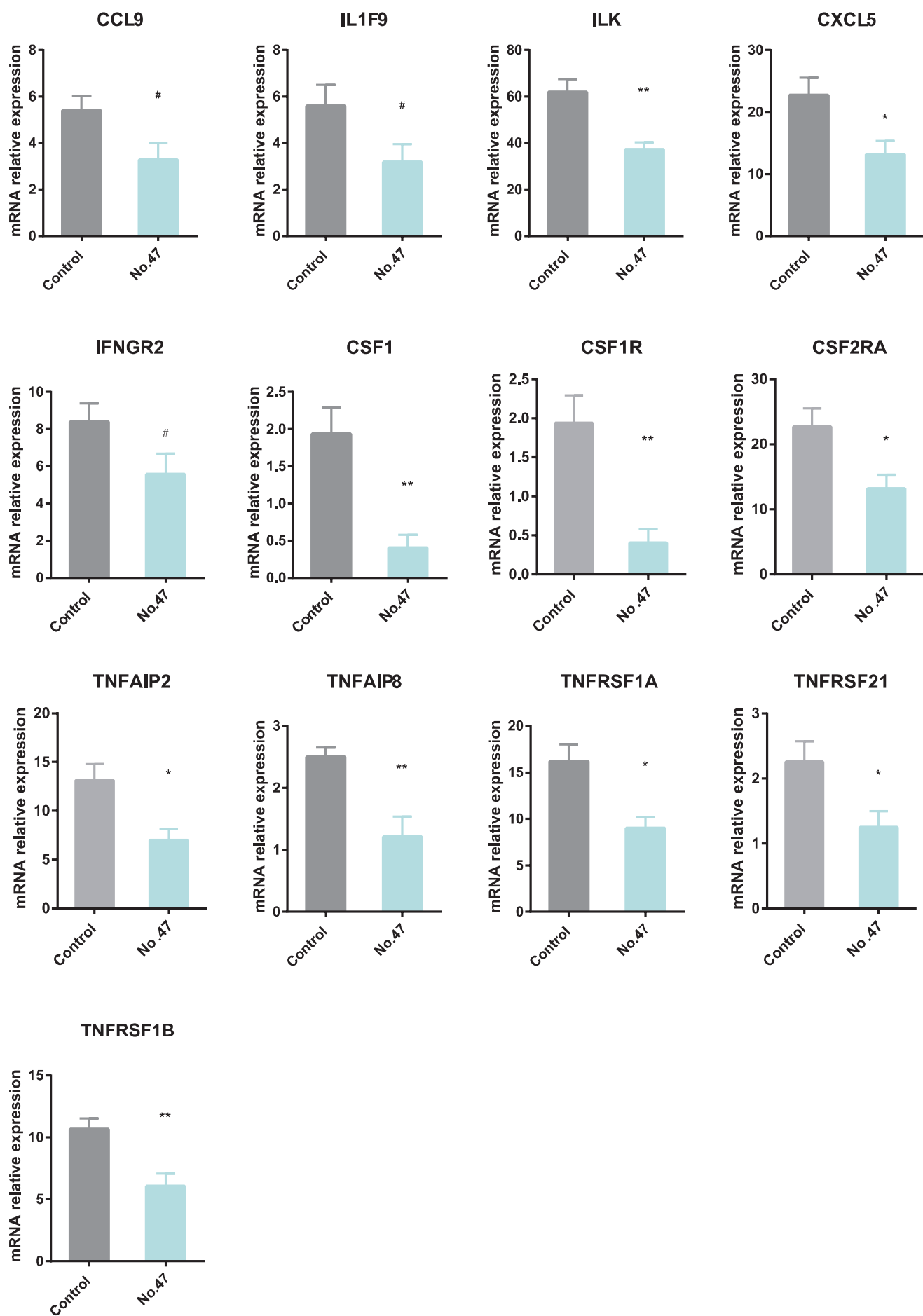

**F**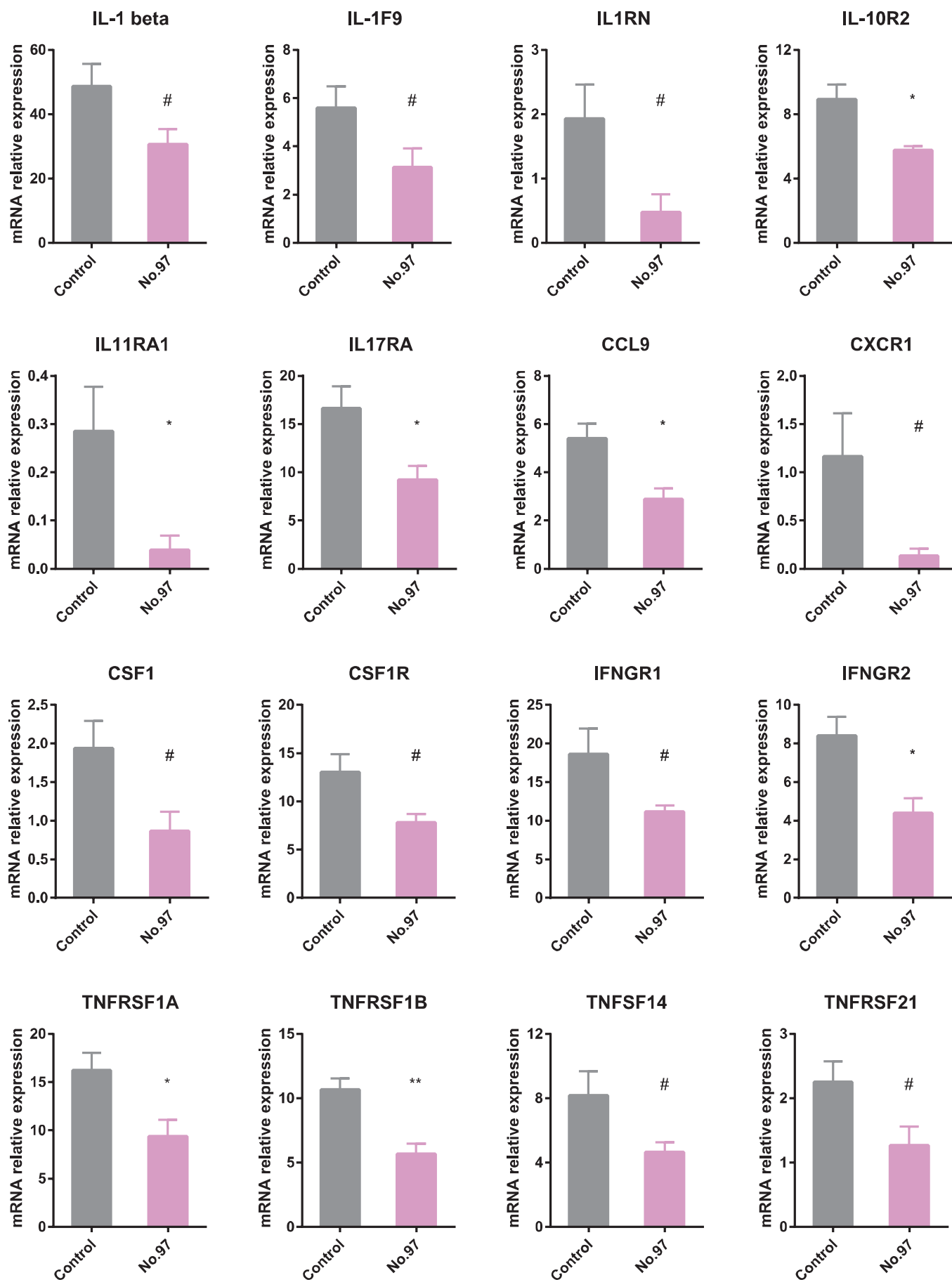

**G**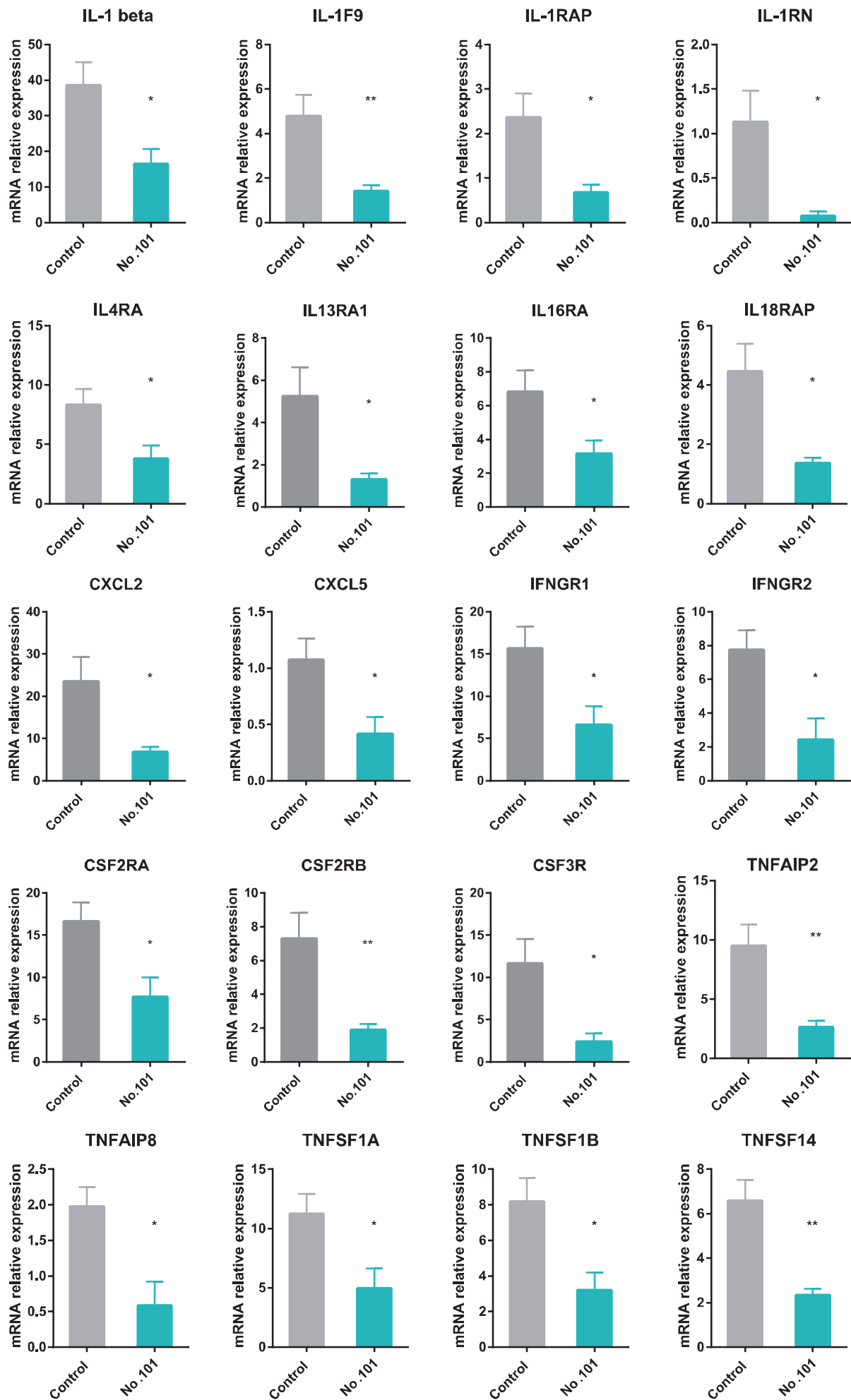

# H

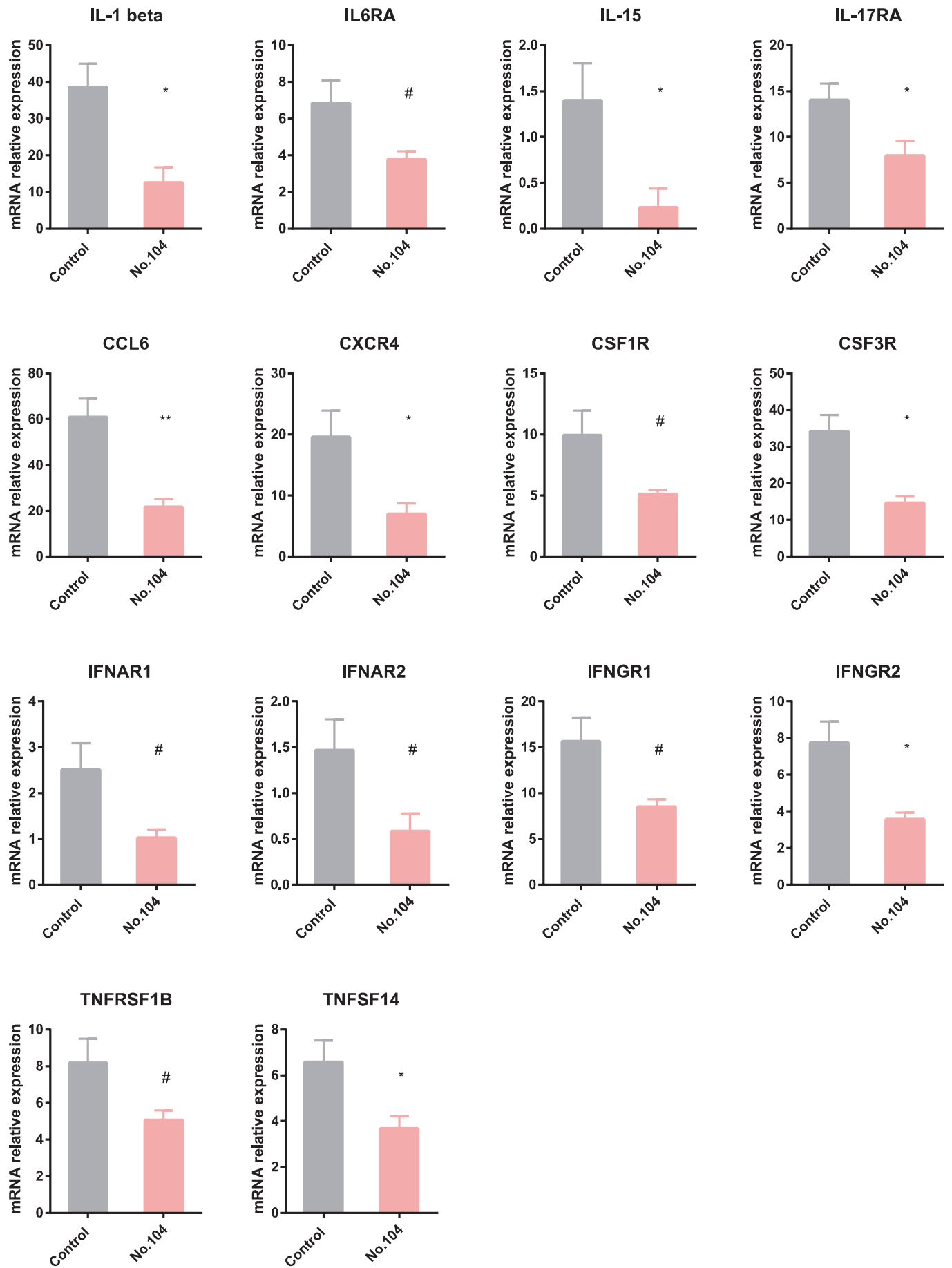

Supplement: FIG S6 [file mSystems.00431-19-sf006.pdf]
